# Genome-Wide Identification and Expression Analysis of the Soybean *GmHSP100* Gene Family in Response to Heat and Salt Stresses

**DOI:** 10.3390/genes17060608

**Published:** 2026-05-27

**Authors:** Bowen Lin, Xinyuan Zhang, Zhiru Yu, Wenjing Zhao, Guanglei Ma, Shuwang Song, Xiaoming Li, Yongbin Zhuang, Jinfei Zhang, Dajian Zhang, Baoyin Chen

**Affiliations:** National Key Laboratory of Wheat Improvement, College of Agronomy, Shandong Agricultural University, Tai’an 271018, China

**Keywords:** soybean, heat shock proteins, ClpB protein, genome-wide analysis, abiotic stress

## Abstract

Background: Heat shock protein 100 (HSP100) is a key molecular chaperone that maintains intracellular proteostasis and enhances plant tolerance. However, the *HSP100* gene family in soybean (*Glycine max*) has not been systematically characterized. Methods: In this study, we performed genome-wide identification and comprehensive analysis of the *GmHSP100* gene family and analyzed their phylogeny, genomic distribution, synteny, protein structures, subcellular localization, promoter cis-elements, and expression patterns under heat and salt stresses via bioinformatics approaches and quantitative real-time PCR (qRT-PCR) validation. Results: Thirteen *GmHSP100* members were identified, which were classified into *CLPB*, *CLPC* and *CLPD* subfamilies. Segmental and whole-genome duplications primarily drove the expansion of this gene family. All encoded proteins possessed conserved AAA+ ATPase domains, with distinct motifs across subfamilies. Most proteins localized to the cytoplasm, while CLPC and CLPD targeted chloroplasts and GmCLPB4 localized to mitochondria. Promoter analysis identified numerous elements associated with light, hormone and stress responses. Expression profiling showed strong tissue specificity and time-dependent stress-treatment induction. Heat stress triggered rapid and strong upregulation of the *GmHSP100s*, whereas salt stress salt stress induced their relatively delayed and sustained expression. Conclusions: These findings reveal the evolutionary conservation and diversification of the *GmHSP100* gene family in soybean, providing a foundational framework for understanding the functions of *GmHSP100* in stress adaptation.

## 1. Introduction

Soybean (*Glycine max*) is one of the most economically important legume crops worldwide, providing abundant vegetable oil, high-quality protein, and raw materials for food processing and animal husbandry [1]. As a major source of plant protein and oil, soybean plays a critical role in global food security and agricultural sustainability. However, frequent extreme climate events, such as high temperature, salinity, drought, and cold, severely restrict soybean growth, development, photosynthetic efficiency, reproductive processes, and yield stability [2]. Among these abiotic stresses, heat stress and salt stress are the two most destructive factors that cause protein denaturation, reactive oxygen species (ROS) accumulation, cellular damage, and metabolic disorders, ultimately leading to significant yield losses [2,3,4,5,6,7,8]. Therefore, dissecting the molecular mechanisms underlying soybean adaptation to heat and salt stress is of great theoretical significance for breeding stress-resistant soybean varieties.

Heat shock proteins (HSPs) are a superfamily of highly conserved molecular chaperones that are rapidly induced under stress conditions [9]. HSP100 (also known as ClpB or HSP101) belongs to the AAA+ ATPase superfamily and functions as a core disaggregase that resolubilizes stress-induced protein aggregates in cooperation with the HSP70–HSP40 chaperone system [10]. By refolding denatured proteins or targeting misfolded proteins for degradation, HSP100 proteins maintain intracellular proteostasis and enhance plant survival in adverse environments [11,12,13,14,15]. Previous studies have demonstrated that *HSP100* genes are essential for acquired thermotolerance and are also involved in salt, drought, and cold stress responses [16,17]. In Arabidopsis, *AtHSP101* is essential for both inherent and acquired thermotolerance [18]. It restores heat-damaged proteins by reactivating stress-induced protein aggregates, and mutants lacking this function exhibit severe sensitivity to heat and cannot survive otherwise non-lethal heat stress after acclimatization [18]. In rice, *OsClpB* is regulated in a stress-specific manner, with *OsClpB-m* being the most heat-inducible variant, while *OsClpD1* effectively complements the thermosensitive phenotype in yeast [19,20,21]. In contrast, wheat *TaCLPB* gene family members display homeolog-specific expression patterns in response to heat stress, indicating functional diversification among the *HSP101/CLPB* gene family [19,20,21].

In legumes, several studies have reported that *HSP100* genes are induced by heat stress and participate in improving pollen viability and photosynthetic systems [22,23,24,25]. However, most studies have focused on individual genes, and a systematic genome-wide analysis of the *GmHSP100* gene family is still lacking. The evolutionary relationships, gene structures, conserved domains, chromosomal localization, duplication events, subcellular functional divergence, and precise expression patterns of *GmHSP100* genes under heat and salt stress remain largely unknown. With the completion of soybean whole-genome sequencing, it is possible to systematically identify and characterize the *GmHSP100* gene family at the genome-wide level [20,26,27,28].

In this study, we identified 13 *GmHSP100* family members from the soybean genome using bioinformatics approaches. We comprehensively analyzed their phylogenetic relationships, chromosomal distribution, gene structures, conserved motifs, protein domains, syntenic relationships, and promoter cis-acting elements. Furthermore, we investigated the tissue-specific expression patterns and dynamic expression profiles under heat and salt stress using transcriptome data and RT-qPCR verification. This study aims to reveal the evolutionary characteristics and potential biological functions of the *GmHSP100* gene family in soybean stress adaptation and provide key candidate genes for molecular breeding of heat- and salt-tolerant soybean cultivars.

## 2. Materials and Methods

### 2.1. Identification of GmHSP100 Family Members in Soybean

The soybean reference genome (Williams 82.a4.v1) and corresponding protein sequences were downloaded from Phytozome 13 (https://phytozome-next.jgi.doe.gov/; accessed on 13 November 2025) [29]. The hidden Markov model (HMM) profiles of HSP100 conserved domains (PF00004, PF02861, PF10431, PF17871) were obtained from the Pfam database (http://pfam.xfam.org/family; accessed on 14 November 2025). HMMER 3.3.2 software was used to search the soybean protein database with an E-value threshold of 1 × 10^−5^. Meanwhile, six Arabidopsis HSP100 protein sequences (At1g74310, At5g15450, At2g25140, At3g48870, At5g51070, At5g50920) [30] were used as queries to perform local BLASTP searches, a process conducted using TBtool (v2.454) [31], against the soybean proteome with the same threshold. The two results were merged, and redundant sequences were manually removed. All candidate proteins were further verified using the NCBI Conserved Domain Database (CDD) (https://www.ncbi.nlm.nih.gov/cdd/; accessed on 14 November 2025) [32] to ensure the presence of complete conserved domains characteristic of the HSP100 family.

### 2.2. Chromosomal Localization and Physicochemical Property Analysis

The chromosomal position information of each *GmHSP100* gene was extracted from the genome GFF3 file and visualized using TBtools (v2.454) [31]. The amino acid sequence length, molecular weight, theoretical isoelectric point (pI), aliphatic index, instability index, and grand average of hydropathicity (GRAVY) of each GmHSP100 protein were calculated using TBtools [31]. Subcellular localization was predicted using the WoLF PSORT (https://wolfpsort.hgc.jp/; accessed on 27 February 2026) [33] online server.

### 2.3. Phylogenetic and Synteny Analysis

Full-length protein sequences of HSP100s from soybean, rice, and Arabidopsis (Table S1) were aligned using ClustalW in MEGA11 (v11.0.13) [34]. A phylogenetic tree based on the neighbor-joining (NJ) approach was generated with 1000 bootstrap replications, and subsequent visualization and annotation were performed using the iTOL online platform. Intra-species and inter-species synteny analyses were performed using MCScanX integrated into TBtools (v2.454) [31]. The collinear relationships were plotted using a Circos diagram.

### 2.4. Gene Structure, Conserved Motif, and Domain Analysis

The exon–intron structures of *GmHSP100* genes were extracted from the GFF3 annotation file and visualized using TBtools (v2.454) [31]. Conserved motifs were identified using the MEME ver 5.5.8 online tools (https://meme-suite.org/meme/tools/meme; accessed on 14 November 2025) with the following parameters: maximum number of motifs = 15, and the others set as default [35]. Conserved domains were annotated using NCBI CDD (https://www.ncbi.nlm.nih.gov/cdd/; accessed on 14 November 2025) [32]. The gene structures, conserved motifs, and domains were integrated and displayed using TBtools (v2.454) [31].

### 2.5. Promoter Cis-Acting Element Analysis

To investigate potential regulatory features, the upstream 2000-bp regions from the translation initiation site of each GmHSP100 member were defined as promoter sequences. Regulatory cis-elements were then predicted using the PlantCARE database (https://bioinformatics.psb.ugent.be/webtools/plantcare/html/; accessed on 16 December 2025) [36], followed by statistical classification and graphical presentation in TBtools (v2.454) [31].

### 2.6. Plant Materials, Stress Treatments, and Sampling

The soybean cultivar Williams 82 was used in this study. Seeds were sterilized and sown in plastic pots filled with a 1:1 mixture of vermiculite and nutrient soil. Seedlings were grown in a growth chamber under 16 h light/8 h dark, 22 °C, 68–73% humidity. At the V1 stage (first trifoliate leaf fully expanded), seedlings were subjected to heat or salt stress. For heat stress treatment, seedlings were exposed to 37 °C, and trifoliate leaves were sampled at 0, 0.5, 1, 2, 6, 12, and 24 h. For salt stress treatment, seedlings at the VC stage (when the unifoliate leaves are half-expanded with separated leaf margins) were placed in a hydroponic system with half-strength Hoagland nutrient solution and allowed to grow until they reached the V1 stage. Subsequently, they were subjected to a treatment of 120 mM NaCl in the same half-strength Hoagland nutrient solution, and root samples were taken at 0, 0.5, 1, 2, 6, 12, and 24 h. All samples were immediately frozen in liquid nitrogen and stored at −80 °C. Three biological replicates were performed for each treatment.

### 2.7. Tissue-Specific Expression Analysis

Transcriptome data (Fragments Per Kilobase of transcript per Million mapped reads (FPKM) values) of different soybean tissues, including root nodule, root, stem, leaf, flower, and seed, were downloaded from the SoyBase database (https://data.soybase.org/Glycine/max/expression/Wm82.gnm4.ann1.expr.Wm82.Sreedasyam_Plott_2023/; accessed on 12 May 2026). The expression data were processed and normalized via Z score method using the ‘data.table’ package in R (v4.5.1). A tissue-specific expression heatmap was generated using the pheatmap package.

### 2.8. Single-Cell Expression Analysis

The single-cell transcriptome dataset used in this study was obtained from the National Genomics Data Center (https://ngdc.cncb.ac.cn/gsa/index.jsp; accessed on 12 May 2026) [37]. The dataset includes single-nucleus RNA sequencing (snRNA-seq) data derived from soybean root, root nodule, and leaf tissues. Gene expression profiles of the *GmHSP100* gene family were extracted from the integrated atlas and further analyzed at the single-cell level. Dot plots were generated to visualize the expression patterns of *GmHSP100* genes across different cell types. In the dot plots, dot size represents the proportion of cells expressing the corresponding gene within each cell cluster, while color intensity indicates the average normalized expression level. All visualization analyses were performed using the Seurat package in R. For functional annotation and cross-study comparison, the Zhonghuang 13 gene models were mapped to their Williams 82 (Wm82.a4.v1) orthologs based on homology relationships. Gene expression quantification was performed using the Zhonghuang 13 reference annotation. Gene names shown in the bubble plots were standardized solely for visualization purposes according to the corresponding W82 orthologous gene IDs, and detailed correspondence is provided in Table S2.

### 2.9. RNA Extraction and RT–qPCR Analysis

Total RNA was prepared from soybean tissues with TRIzon reagent (Cowin Biotech, Beijing, China). RNA integrity and concentration were examined using a NanoDrop One/OneC instrument (Thermo Fisher Scientific, Waltham, MA, USA). First-strand cDNA was synthesized using HiScript II Q RT SuperMix (+gDNA wiper) (Vazyme, Nanjing, China). Gene-specific primers were designed using NCBI Primer-BLAST and are listed in Table 1. The soybean Actin gene (*Glyma.18G290800*) was used as the internal reference.

Quantitative PCR analysis was conducted with a StepOnePlus detection system (Thermo Fisher Scientific, Waltham, MA, USA) using ChamQ Universal SYBR qPCR Master Mix (Vazyme, Nanjing, China). PCR amplification was initiated at 95 °C for 30 s, followed by 40 successive cycles consisting of denaturation at 95 °C for 10 s and annealing/extension at 60 °C for 30 s. A dissociation curve procedure was subsequently included to confirm amplification specificity. Transcript abundance was quantified according to the comparative Ct approach 2^(−ΔΔCt)^ [38]. Three parallel technical repetitions were conducted for every biological sample.

## 3. Results

### 3.1. Identification and Physicochemical Characterization of GmHSP100 Genes

**Figure 1:**
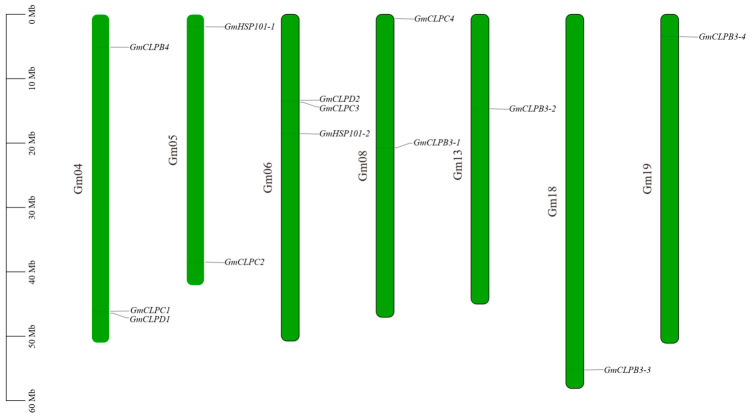
Chromosomal localization of *GmHSP100* family members in soybean. The *GmHSP100* family members are distributed across seven chromosomes, namely Gm04, Gm05, Gm06, Gm08, Gm13, Gm18, and Gm19. Specifically, *GmCLPB4*, *GmCLPC1*, and *GmCLPD1* are located on chromosome Gm04; *GmHSP101-1* and *GmCLPC2* are located on Gm05; *GmCLPD2*, *GmCLPC3*, and *GmHSP101*-2 are located on Gm06; *GmCLPC4* and *GmCLPB3-1* are located on Gm08; *GmCLPB3-2* is located on Gm13; *GmCLPB3-3* is located on Gm18; and *GmCLPB3-4* is located on Gm19. The numbers on the left side of the figure represent chromosome lengths, and the precise positions of each gene on the chromosomes are indicated.

A total of 13 non-redundant *GmHSP100* genes were identified from the soybean genome and named *GmCLPB3-1*, *GmCLPB3-1*, *GmCLPB3-4*, *GmCLPB4*, *GmHSP101-1*, *GmHSP101-2*, *GmCLPD1*, *GmCLPD2*, *GmCLPC1*, *GmCLPC2*, *GmCLPC3*, and *GmCLPC4* according to their phylogenetic relationships and homology with Arabidopsis (Table 2). These genes were unevenly distributed on seven chromosomes (Figure 1). Most genes were located near the proximal or distal regions of the chromosomes, with the exceptions being *GmCLPD2* and *GmCLPC3*. Chromosomes 4 and 6 contained the largest numbers of genes, each harboring three *HSP100* genes. Chromosomes 5 and 8 each contained two genes. The rest of the chromosomes contained only one gene.

The amino-acid sequence length, molecular mass, isoelectric point, instability index, and subcellular localization of the soybean GmHSP100 proteins were systematically analyzed based on predicted physicochemical properties (Table 2). The amino acid sequence lengths of GmHSP100 proteins ranged from 753 aa to 978 aa, with molecular weights from 83.39 kDa to 110.20 kDa, consistent with the structural features of typical HSP100/ClpB-type AAA+ ATPase proteins [39]. The theoretical isoelectric point (pI) values varied from 5.93 (acidic) to 8.81 (basic). Most members were weakly acidic or near neutral, whereas GmCLPD1 and GmCLPD2 showed basic protein characteristics. The instability index ranged from 34.90 to 44.29, and six proteins (GmCLPB3-2, GmCLPB3-4, GmHSP101-1, GmHSP101-2, GmCLPD1, and GmCLPD2) were stable (instability index < 40). All GmHSP100 proteins were hydrophilic with high aliphatic indices, indicating strong thermal stability. The results for GmCLPC1, GmCLPC2, and GmCLPC4 were slightly above 40, showing that they may be less stable under in vitro conditions.

Subcellular localization prediction indicated that CLP/HSP100 family members are mainly localized to chloroplasts and the cytoplasm (Table 2). Specifically, GmCLPB3-1, GmCLPB3-3, GmCLPB3-2, GmCLPB3-4, GmCLPD1, GmCLPD2, GmCLPC1, GmCLPC3, GmCLPC2, and GmCLPC4 members were predicted to localize mainly to chloroplasts; GmHSP101-1 and GmHSP101-2 were localized to the cytoplasm; and GmCLPB4 was predicted to localize to mitochondria. This distribution is consistent with the functional characteristics of different Clp/HSP100 subfamilies in maintaining protein folding and homeostasis in distinct cellular compartments. Overall, soybean GmHSP100 proteins were highly consistent in sequence length, physicochemical properties, and subcellular localization, whereas the various subfamilies showed clear divergence in compartmental distribution, suggesting specialized roles in protein quality control and stress responses across organelles.

### 3.2. Phylogenetic Analysis and Classification of the Soybean HSP100 Family

**Figure 2:**
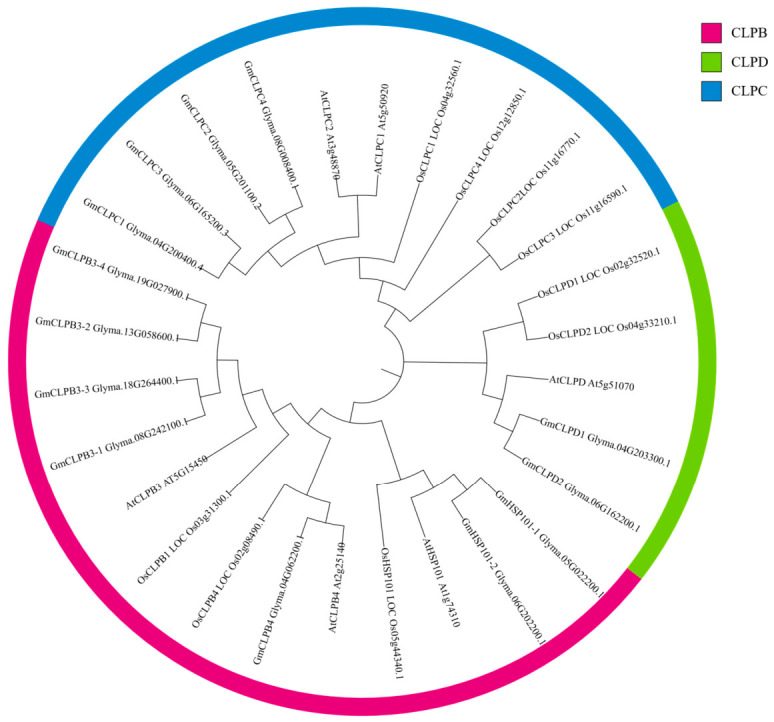
Phylogenetic analysis of the HSP100 family. The figure illustrates the phylogenetic relationships among HSP100 proteins from multiple species, including *Arabidopsis thaliana* (At), *Glycine max* (Gm), and *Oryza sativa* (Os). Different colors represent distinct groups of HSP100 proteins.

A phylogenetic tree was constructed using the full-length amino acid sequences derived from 13 *GmHSP100* genes in soybean, 9 *OsHSP100* genes in rice, and 6 *AtHSP100* genes in *Arabidopsis thaliana* (Figure 2). Phylogenetic analysis demonstrated that the GmHSP100 protein family could be divided into three distinct subfamilies, designated *CLPB*, *CLPC*, and *CLPD*. Among these, the *CLPB* subfamily represented the largest clade, containing seven members, followed by the *CLPC* subfamily with four members and the *CLPD* subfamily with two members.

**Figure 3:**
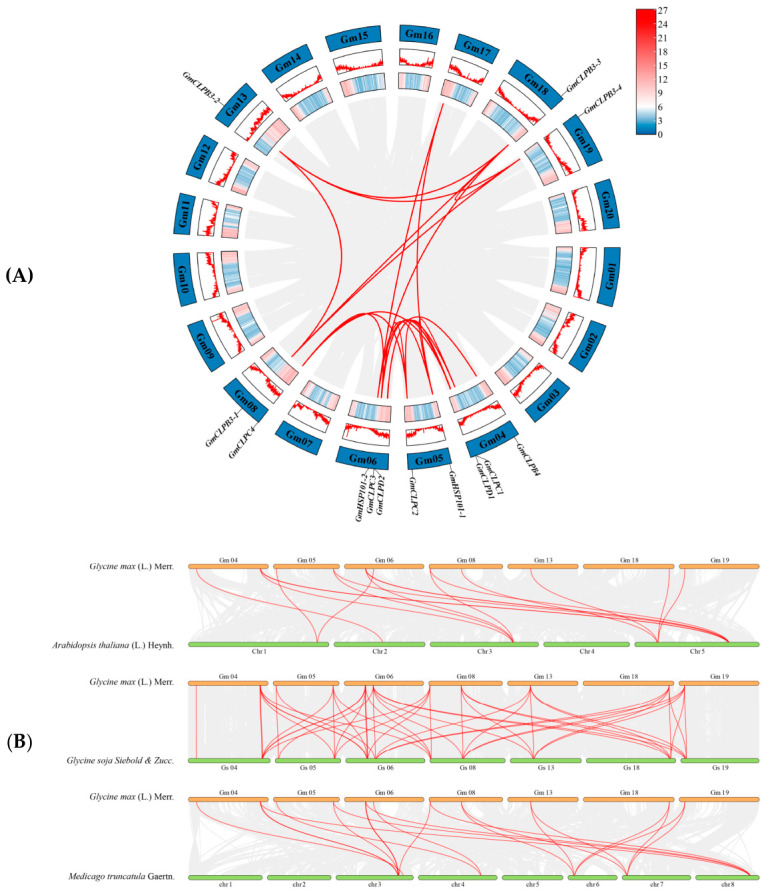
Synteny and cross-species collinearity analysis of the *GmHSP100* gene family. (**A**) Collinearity analysis of *GmHSP100* genes within the *Glycine max* genome. The number and length of chromosomes are shown, and red lines indicate collinear relationships between genes. (**B**) Cross-species collinearity analysis of *HSP100* genes among different genomes. Chromosomes of *Glycine max* are shown at the top, while those of *Arabidopsis thaliana*, *Glycine soja*, and *Medicago truncatula* are displayed below. Red lines represent collinear relationships between *GmHSP100* genes and their homologs in these species.

To further explore the evolutionary origin, duplication history, and interspecific conservation of the *GmHSP100* gene family, both intraspecific and interspecific synteny analyses were performed (Figure 3). Intraspecific synteny analysis revealed extensive syntenic relationships among *GmHSP100* genes distributed across multiple chromosomes (Figure 3A). Notably, the majority of syntenic pairs were mapped to interchromosomal regions rather than adjacent intrachromosomal loci, strongly indicating that segmental duplication and whole-genome duplication—rather than tandem duplication—served as the predominant evolutionary forces driving the expansion and diversification of the *GmHSP100* family in soybean.

Interspecific synteny analysis further revealed highly conserved collinearity (Figure 3B). Abundant one-to-one and one-to-many orthologous pairs were identified between soybean and wild soybean (*Glycine soja*), suggesting that the *HSP100* gene family maintained high conservation in both gene structure and chromosomal localization during soybean domestication. Synteny comparison between soybean and *Medicago truncatula* also revealed that multiple *GmHSP100* genes were located within highly conserved syntenic blocks, supporting the notable evolutionary conservation of the *HSP100* family across legume species. In contrast, only a limited number of syntenic *HSP100* gene pairs were detected between soybean and *Arabidopsis thaliana*, implying significant lineage-specific divergence of the *HSP100* family following the evolutionary separation of legumes and Brassicaceae.

### 3.3. Conserved Motif Identification, Functional Domains, and Gene Structure Analysis of the GmHSP100 Family

**Figure 4:**
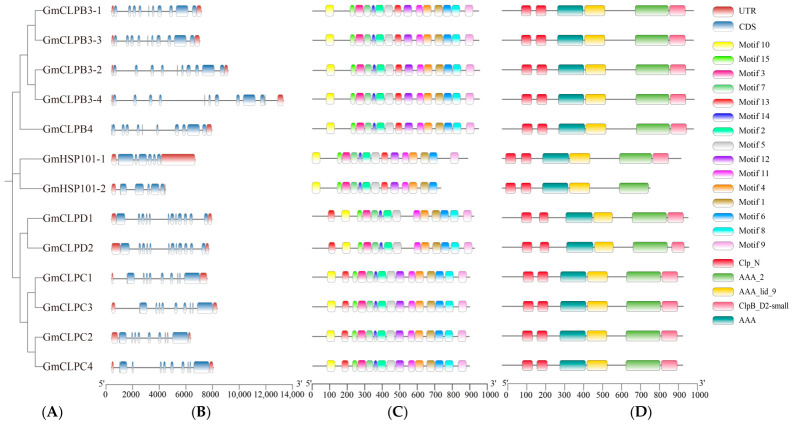
Phylogenetic relationships, gene structures, conserved motifs, and domain architecture of the soybean *HSP100* gene family. (**A**) Phylogenetic analysis. (**B**) Gene structure analysis: blue boxes represent coding sequences (CDS), white boxes represent untranslated regions (UTRs), and thin lines indicate introns. (**C**) Distribution of conserved motifs: fifteen conserved motifs are identified in the family proteins, with different colors representing different motifs. (**D**) Protein domain architecture: the domain composition of soybean HSP100 proteins, with different colors indicating different types of domains.

To comprehensively characterize the structural conservation and functional divergence of *GmHSP100* family members, we systematically analyzed conserved motifs, functional domains, and exon–intron architectures (Figure 4). A comparison of gene structures revealed substantial variation in genomic length and exon–intron organization among *GmHSP100* genes (Figure 4A,B). Gene sizes spanned several kilobases, up to approximately 14 kb, with several genes harboring large intronic regions (Figure 4B). In contrast, exon number and arrangement were highly conserved within each subfamily, indicative of strong subfamily-specific structural conservation and consistent evolutionary trajectories among closely related members (Figure 4A,B).

A total of 15 distinct conserved motifs were identified across the *GmHSP100* family (Figure 4C). All members share a set of core conserved motifs, including Motif 10, Motif 15, Motif 3, Motif 7, Motif 13, Motif 14, Motif 2, Motif 5, Motif 11, Motif 4, Motif 1, and Motif 6. Most members also contain Motif 12, Motif 8, and Motif 9. Notably, the position of Motif 13 differs between the CLPD and CLPC subfamilies compared to the CLPB subfamily. The CLPD subfamily lacks Motif 12, while the CLPB subfamily members GmHSP101-1 and GmHSP101-2 lack Motif 8, and GmHSP101-2 additionally lacks Motif 9. These findings suggest a potential role in subfamily-specific functional divergence. Overall, these observations support a modular structural organization: the universally shared motifs form a core functional scaffold essential for fundamental chaperone activity, while subfamily-restricted or partially distributed motifs likely contribute to functional diversification, regulatory specificity, and environmental adaptation.

Domain architecture analysis further confirmed high overall conservation within the GmHSP100 family (Figure 4D). All proteins contained the canonical and ordered domain combination of Clp_N, AAA, AAA_lid_9, and AAA_2, strongly implying conserved ATP-dependent chaperone function across the family. Further comparison revealed that, with the sole exception of GmHSP101-2, all members carried a diagnostic ClpB_D2-small domain at the C-terminal region. GmHSP101-2 lacked this domain and exhibited a noticeably shorter protein length, suggesting partial structural truncation or simplification during evolution.

Taking these findings together, the GmHSP100 family displays high conservation in core domain composition and order, while subtle structural variations, including domain loss and differences in protein length, have emerged during evolution. These changes occurred without compromising core molecular function and likely served as the structural basis for the functional diversification observed among subfamilies.

### 3.4. Cis-Acting Element Analysis of GmHSP100 Promoters

**Figure 5:**
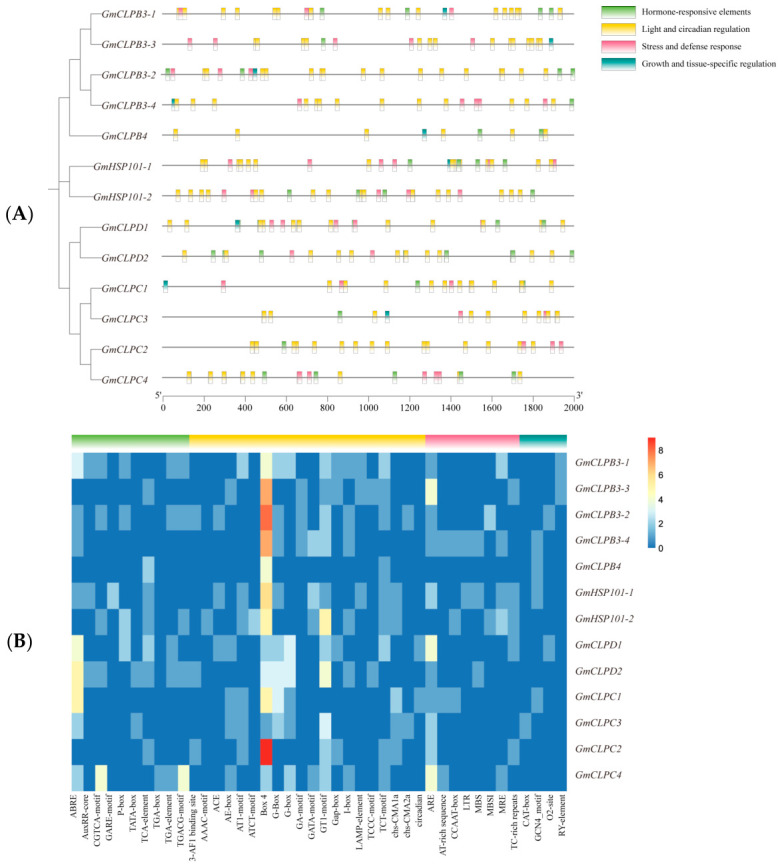
Cis-acting element analysis of promoters in the *GmHSP100* gene family. (**A**) Distribution of cis-acting elements: the number and distribution of four categories of key cis-acting elements in the promoter regions of each gene are shown. Different colors represent different functional types of elements. The *x*-axis indicates the relative positions of elements in the promoter sequences (in base pairs). (**B**) Heatmap of cis-acting element abundance: the y-axis represents the names of *GmHSP100* family genes, and the *x*-axis represents the names of cis-acting elements. The color gradient indicates the abundance of specific elements in each gene.

As shown in Figure 5A, the identified cis-acting elements were classified into four major functional categories, including light-responsive elements, growth and development-related elements, hormone-responsive elements, and stress-responsive elements. The composition and abundance of these elements varied considerably among different *GmHSP100* genes (Figure 5B). Light-responsive elements were the most abundant group, among which Box 4 and GT1-motif were the predominant types. In contrast, elements related to growth and developmental regulation were the least abundant. For hormone responsiveness, most elements were associated with abscisic acid (ABA) and methyl jasmonate (MeJA) signaling pathways, with ABRE being the predominant ABA-responsive element detected in nearly all promoters. For stress responsiveness, the main types included ARE, LTR, and MBS, among which ARE was the most abundant, followed by MBS. Collectively, these results indicate that *GmHSP100* genes are subject to complex transcriptional regulation and may participate extensively in light signal transduction, hormone signaling pathways, multiple abiotic stress responses, and various growth and developmental processes in soybean.

### 3.5. Tissue-Specific Expression Profiles of the GmHSP100 Family

**Figure 6:**
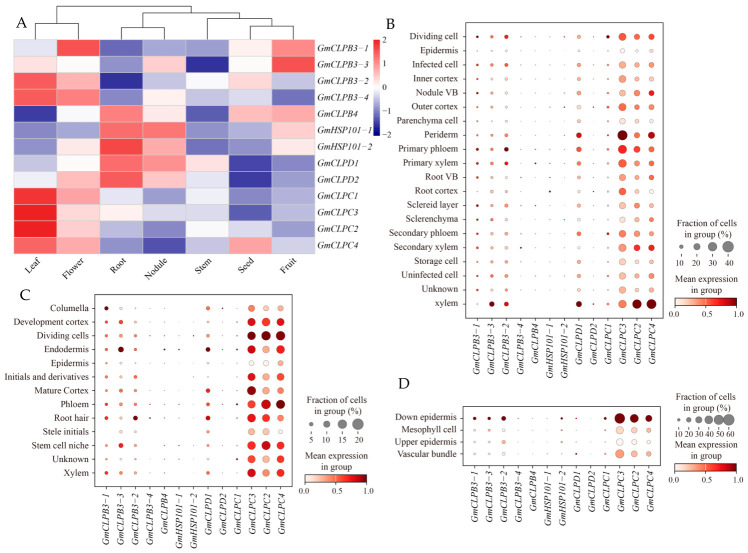
Tissue-specific and single-cell expression patterns of the *GmHSP100* gene family in soybean. (**A**) Heatmap showing the expression profiles of *GmHSP100* family genes across different soybean tissues, including leaf, flower, root, nodule, stem, seed, and fruit. Red and blue indicate relatively high and low expression levels, respectively. Hierarchical clustering analysis was performed based on gene expression patterns. (**B**–**D**) Dot plots showing the single-cell expression patterns of GmHSP100 family genes in soybean root (**B**), nodule (**C**), and leaf (**D**) tissues. Dot size represents the proportion of cells expressing the corresponding gene within each cell type, while color intensity indicates the average expression level. Different cell types are labeled on the y-axis, and gene IDs are shown on the x-axis.

*GmHSP100* family members exhibited significantly divergent expression patterns across different tissues (Figure 6A). Cluster analysis revealed that leaves and flowers shared highly similar expression profiles, and the roots and nodules clustered together, with seeds and pods also showing considerable similarity, suggesting that these tissues may share comparable functional requirements and regulatory mechanisms. Within the CLPB subfamily, *GmCLPB3-1*, *GmCLPB3-2*, and *GmCLPB3-4* showed relatively high expression in leaves and flowers but low expression in roots and stems. *GmCLPB3-1*, *GmCLPB3-3,* and *GmCLPB4* were highly expressed in pods but showed relatively low expression in stems. In addition, *GmCLPB4*, *GmHSP101-1*, and *GmHSP101-2* were highly expressed in roots, nodules, and pods, while exhibiting low expression in leaves, indicating potential functional divergence among CLPB members at the tissue level. CLPD subfamily members *GmCLPD1* and *GmCLPD2* showed relatively high expression in roots and nodules, whereas their expression levels were markedly reduced in seeds and pods. CLPC subfamily members were generally highly expressed in leaves and flowers, with *GmCLPC1*, *GmCLPC2*, and *GmCLPC3* showing particularly strong expression in these tissues, while exhibiting relatively low expression in nodules, seeds, and pods. In contrast, *GmCLPC4* showed high expression in leaves and flowers as well as relatively elevated expression in seeds, but low expression in nodules. Overall, *GmHSP100* family members displayed pronounced tissue-specific expression patterns, suggesting that they may play diverse and tissue-specific roles in soybean growth and development as well as in protein quality control processes.

Furthermore, single-cell transcriptome data were used to dissect the cell type-specific expression patterns of these genes in roots, nodules, and leaves (Figure 6B–D). In nodules (Figure 6B), *GmCLPC1* showed highly specific expression in meristematic cells, whereas most genes were predominantly enriched in vascular tissues such as xylem and primary phloem, with generally low expression in infected cells. In roots (Figure 6C), *GmCLPB3-3* and *GmCLPD1* exhibited strong endodermis-specific expression, while GmCLPB3-4 showed specific high expression in root hair cells. In addition, most genes showed relatively higher expression in the dividing cells and developing cortex, suggesting potential roles in root growth and development. In leaves (Figure 6D), these genes were mainly enriched in the lower epidermis, with relatively low expression in other cell types. Notably, *GmCLPC2*, *GmCLPC3*, and *GmCLPC4* exhibited broad and stable expression across most cell types in nodules, roots, and leaves, suggesting that they may function as core “housekeeping” genes involved in maintaining intracellular protein homeostasis and participating in fundamental processes of nodule development and functional maintenance.

### 3.6. Expression Analysis of GmHSP100 Under Heat and Salt Stress

To further dissect the potential functions of the *GmHSP100* family in abiotic stress responses, we first analyzed promoter cis-elements and found that most *GmHSP100* genes harbor abundant ABA-responsive elements (ABREs) and various stress-related regulatory motifs, implying broad involvement in stress signaling and tolerance. Accordingly, soybean seedlings were exposed to heat stress and salt stress, and transcript changes in the 13 *GmHSP100* genes were monitored at 0, 0.5, 1, 2, 6, 12, and 24 h. Under our experimental conditions, *GmCLPB3-3* exhibited no detectable expression at any time point.

**Figure 7:**
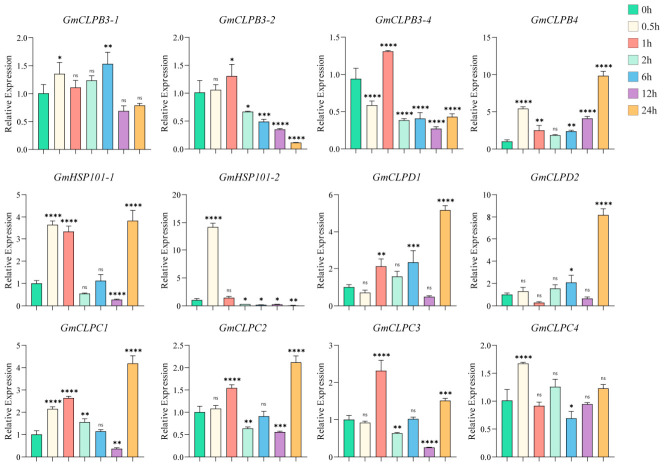
Temporal expression patterns of *GmHSP100* genes under heat stress. The relative expression levels of 12 genes were measured at 0 h, 0.5 h, 1 h, 2 h, 6 h, 12 h, and 24 h under heat stress. Different colors represent different time points. Data are presented as the mean ± standard deviation (SD) of three independent biological replicates. Statistical analysis was performed using one-way ANOVA with Dunnett’s multiple comparison test, comparing all experimental groups to the control group. * *p* < 0.05, ** p < 0.01, *** *p* < 0.001, **** *p* < 0.0001; ns, not significant.

Under heat stress, the *GmHSP100* genes exhibited a clear time-dependent expression pattern, with pronounced subfamily- and member-specific differences (Figure 7). Within the *CLPB* subfamily, induction patterns varied considerably. The genes *GmCLPB3-1*, *GmCLPB3-2*, *GmCLPB3-4*, *GmCLPB4*, *GmHSP101-1*, and *GmHSP101-2* showed low basal expression levels under control conditions (0 h). Following heat stress, all genes were rapidly upregulated, reaching peak expression at 0.5–1 h and then gradually declining as treatment time progressed. Notably, *GmHSP101-1* and *GmHSP101-2* displayed significantly higher induction levels than other *CLPB* members, representing the strongest responders within this subfamily and supporting their critical roles in heat stress tolerance. In the *CLPD* subfamily, *GmCLPD1* and *GmCLPD2* responded rapidly and strongly to heat stress. Significant upregulation was observed as early as 0.5 h, showing an overall pattern of rapid increase, sustained high expression, and gradual decline. The expression profiles of these two genes were highly similar, with *GmCLPD2* exhibiting slightly higher expression levels than *GmCLPD1*. Notably, both genes were strongly re-induced at 24 h. These patterns suggest that *CLPD* genes are involved in plastid protein quality control and cellular homeostasis under heat stress. For the *CLPC* subfamily, *GmCLPC1*, *GmCLPC2*, *GmCLPC3*, and *GmCLPC4* exhibited lower induction levels compared with the *CLPB* and *CLPD* subfamilies. Their expression peaked at 0.5–2 h, followed by a gradual decline, while remaining higher than control levels at 24 h. The expression trends among members were similar, with *GmCLPC1* showing the highest induction and *GmCLPC3* relatively lower expression. Overall, the *CLPB*, *CLPC*, and *CLPD* subfamilies in soybean all participate in the early response to heat stress, characterized by rapid upregulation at early stages and strong heat responsiveness. Notably, *GmCLPB4*, *GmHSP101-1*, *GmCLPD1*, *GmCLPD2*, and *GmCLPC1* were strongly induced at 24 h, with CLPB (including HSP100) and CLPD representing highly responsive subfamilies, while CLPC represents a moderately responsive subfamily.

**Figure 8:**
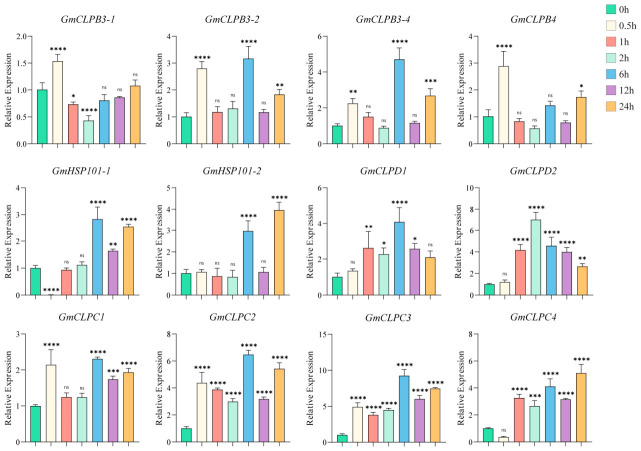
Temporal expression patterns of *GmHSP100* genes under salt stress. The relative expression levels of 12 genes were measured at 0 h, 0.5 h, 1 h, 2 h, 6 h, 12 h, and 24 h under salt stress. Different colors represent different time points. Data are presented as the mean ± standard deviation (SD) of three independent biological replicates. Statistical analysis was performed using one-way ANOVA with Dunnett’s multiple comparison test, comparing all experimental groups to the control group. * *p* < 0.05, ** *p* < 0.01, *** *p* < 0.001, **** *p* < 0.0001; ns, not significant.

Under salt stress, *GmHSP100* genes also exhibited clear time-dependent transcriptional changes, although their specific response patterns differed from those under heat stress (Figure 8). Within the *CLPB* subfamily, *GmCLPB3-1*, *GmCLPB3-2*, *GmCLPB3-4*, and *GmCLPB4* displayed an induction pattern characterized by rapid upregulation at early stages, followed by a gradual decline, and a subsequent increase again at middle to late stages. Expression peaks were mainly observed at 0.5 h and 6 h. *GmHSP101-1* and *GmHSP101-2* were also induced by salt stress, with peak expression primarily occurring at the middle to late stages (6–24 h). Among these, *GmCLPB3-2*, *GmCLPB3-4*, and *GmHSP101-2* exhibited the strongest responses within this subfamily. In the *CLPD* subfamily, *GmCLPD1* and *GmCLPD2* both showed a sustained induction pattern under salt stress. Their expression levels increased rapidly from 1 to 6 h, followed by a gradual decline after 12 h, while remaining higher than control levels at 24 h. The overall induction intensity of *GmCLPD2* was slightly higher than that of *GmCLPD1*, and their expression profiles were highly consistent. Members of the *CLPC* subfamily also exhibited pronounced salt responsiveness. *GmCLPC2* and *GmCLPC3* reached relatively high expression levels at 6 h, whereas *GmCLPC4* peaked at 24 h. In contrast, *GmCLPC1* was upregulated at early stages and maintained relatively high expression throughout the treatment period. In brief, the three subfamilies of the *GmHSP100* family are all involved in the early response to salt stress, showing a similar pattern to their response to heat stress. The *CLPC* subfamily shows the most prominent response, the *CLPD* subfamily exhibits sustained high expression, and the *CLPB* subfamily is characterized mainly by transient and rapid induction, indicating functional divergence among subfamilies in soybean salt stress tolerance.

## 4. Discussion

### 4.1. Evolutionary Expansion and Structural Conservation of the GmHSP100 Family

The plant HSP100 family belongs to the evolutionarily conserved AAA+ ATPase superfamily and functions as a core molecular chaperone system involved in protein disaggregation and proteostasis maintenance [9,10]. Its essential roles in abiotic stress tolerance have been well documented in Arabidopsis, rice, and other model plant systems [40,41]. In this study, we systematically identified 13 *GmHSP100* family members from the soybean genome at a whole-genome scale (Figure 1). This number is considerably higher than the six members reported in *Arabidopsis* and nine in rice, indicating lineage-specific expansion of the *HSP100* family in soybean. Phylogenetic analysis clearly classified the *GmHSP100* family into three distinct subfamilies, *CLPB*, *CLPC*, and *CLPD* (Figure 2). This classification is highly consistent with the evolutionary grouping of HSP100 proteins in wheat and other plant species [20], supporting the broad evolutionary conservation of the *HSP100* gene family across both monocotyledonous and dicotyledonous plants. Chromosomal distribution and synteny analyses further revealed that segmental duplication and whole-genome duplication were the predominant evolutionary forces driving the expansion of the *GmHSP100* family. The *GmHSP100* family exhibits a typical evolutionary pattern of “core structural conservation with subfamily-specific diversification”, in which the functional stability of the conserved AAA+ ATPase core domain is strictly maintained, while local structural variations accumulate to enable functional innovation and adaptive divergence [42]. This model is consistent with the evolutionary expansion mechanisms observed for numerous stress-responsive gene families in soybean [43]. Furthermore, the high level of syntenic conservation detected between soybean and wild soybean (*Glycine soja*), as well as between soybean and *Medicago truncatula* (Figure 3B), reinforces the conclusion that the *HSP100* family performs conserved, indispensable functions during legume evolution and stress adaptation.

### 4.2. Functional Divergence of the GmHSP100 Family Based on Subcellular Localization

The maintenance of intracellular proteostasis during stress-induced protein unfolding, aggregation, and disaggregation relies on the coordinated function of multiple molecular chaperone systems [44]. Protein disaggregation and refolding are not executed by individual chaperones, but rather mediated by the collaborative HSP70–HSP100 chaperone system [45,46]. In this pathway, HSP70 initially recognizes and binds misfolded or aggregated substrates, then recruits and activates HSP100, which uses ATP hydrolysis to drive the disaggregation and refolding of damaged proteins, thereby preserving cellular protein homeostasis [15,47].

In plants, the HSP100 family constitutes a core molecular basis for acquired thermotolerance and broad abiotic stress resistance. For instance, a 101 kD HSP from soybean can functionally complement an *HSP104*-deficient yeast mutant, and *Arabidopsis* HSP101 has been verified to cooperate with small heat-shock proteins to protect cellular proteins from stress-induced damage [22,48]. Based on the conserved domain architecture and phylogenetic characteristics of *GmHSP100* family members identified in this study (Figure 4), we infer that this family plays indispensable roles in protein quality control during soybean adaptation to heat and salt stress.

Notably, subcellular localization prediction revealed clear compartmentalized distribution patterns among *GmHSP100* members (Table 2). *GmHSP101-1* and *GmHSP101-2* were predicted to localize predominantly to the cytosol, whereas most members of the CLPC and CLPD subfamilies were preferentially targeted to chloroplasts (Table 2). These distinct localization patterns imply that *GmHSP100* genes maintain cellular proteostasis via spatially specialized functional divergence: cytosolic isoforms likely mediate rapid responses to heat stress and efficient repair of denatured proteins in the cytoplasm, whereas chloroplast-localized members may participate in the assembly, stability, and repair of photosynthetic protein complexes [49,50], thereby safeguarding photosynthetic efficiency under stress. Furthermore, the unique predicted mitochondrial localization of *GmCLPB4* extends the functional scope of the *GmHSP100* family to mitochondrial protein homeostasis. These findings suggest that the *GmHSP100* family establishes a multi-compartment, finely tuned protein quality control network spanning the cytosol, chloroplasts, and mitochondria, which collectively enhances stress resilience and adaptive capacity in soybean [51].

### 4.3. Differences in Signaling Pathways and Regulatory Features Between Heat and Salt Stress Responses

Promoter cis-element analysis revealed that *GmHSP100* promoters are significantly enriched with diverse stress-responsive elements and hormone-responsive elements (Figure 5). This regulatory architecture is highly consistent with the promoter features observed in other stress-related gene families in soybean, such as the *GmHsp70* [52] and *GmWRKY* [53] families, providing a molecular basis for the ability of *GmHSP100* genes to integrate stress and hormonal signals during soybean adaptation to adverse environments.

In the present study, *GmHSP100* genes exhibited markedly distinct transcriptional dynamics under heat and salt stress conditions (Figure 7 and Figure 8). Heat stress induced rapid and strong transcriptional activation, particularly during the early response phase (0.5–1 h), which is consistent with the classical heat shock factor (HSF)-dependent regulatory pathway [54]. Such rapid induction likely reflects an immediate cellular defense mechanism against acute proteotoxic stress caused by extensive protein unfolding and aggregation under high-temperature conditions. In contrast, salt stress triggered a relatively moderate but more sustained transcriptional response. Most genes gradually reached peak expression around 6 h and maintained elevated expression during later stages, suggesting that salt stress responses may primarily represent adaptive and acclimatory processes associated with prolonged osmotic imbalance, ion toxicity, and secondary metabolic disturbances [55,56].

Notably, the rapid and strong upregulation of *GmHSP101-1* and *GmHSP101-2* during the early stage of heat stress supports their proposed roles as emergency disaggregase chaperones involved in rapid protein repair and restoration of proteostasis [57,58]. By comparison, members of the *CLPC* and *CLPD* subfamilies maintained relatively stable or sustained expression during the middle and late stages under both heat and salt stress conditions, implying potential functions in long-term protein quality control, chloroplast protein turnover, and stress adaptation processes, possibly through ABA-mediated signaling pathways.

It should also be noted that the RT–qPCR analyses in this study were performed using bulk tissues, which may mask cell-type-specific transcriptional responses [59]. Given the pronounced spatial heterogeneity revealed by the single-cell transcriptomic analysis, future studies integrating cell-type-specific or spatial transcriptomic approaches will be valuable for dissecting the precise regulatory roles of *GmHSP100* genes during stress adaptation.

### 4.4. Prioritization of Candidate Genes and Future Perspectives

Based on comprehensive evidence from tissue specificity, subcellular localization, and stress-responsive expression profiles, we identified five high-priority candidate genes for subsequent expression validation, including *GmHSP101-2*, *GmCLPB4*, *GmCLPC2*, *GmCLPC3*, and *GmCLPD1*. These genes were significantly induced under both heat and salt stress conditions and exhibited relatively high expression in key tissues, including leaves, flowers, roots, and nodules, suggesting that they may contribute to the maintenance of protein homeostasis during reproductive development and photosynthesis, thereby supporting yield stability under stress conditions (Figure 6). In addition, single-cell transcriptome analysis further revealed cell type-specific expression patterns of these genes in roots, nodules, and leaves. For instance, *GmCLPD1* was significantly enriched in root endodermal cells, whereas *GmCLPC2* and *GmCLPC3* exhibited broad and relatively stable expression across multiple cell types in different tissues. Previous studies have shown that plant *Clp* genes generally display wide tissue expression patterns, in which ClpC is considered a constitutively expressed molecular chaperone that plays essential roles in normal plant growth, development, and chloroplast function maintenance [60]. Moreover, the Clp protease complex is a core component of the chloroplast protein homeostasis and quality control network, playing critical roles in protein folding, degradation, and overall proteostasis maintenance [61]. Therefore, GmCLPC2 and GmCLPC3 may play important roles in basal plastid protein quality control and cellular homeostasis maintenance.

Notably, although single-cell transcriptomic technology was introduced in this study, thereby significantly improving cellular-level resolution, this approach still has inherent limitations. In particular, the process of single-cell isolation may interfere with intercellular communication and alter the spatial architecture of cells and nuclei, making it difficult to fully reflect the true state of complex biological systems that rely on cell–cell interactions [59]. Therefore, future studies integrating interactomics analyses may help to more comprehensively elucidate the underlying regulatory networks and intercellular coordination mechanisms involved in plant stress responses. In addition, *GmCLPB3-3* showed no detectable expression under any tested condition, which may be attributed to highly specific spatial–temporal expression patterns or untested stress conditions [62]. This gene warrants further investigation using expanded tissue panels and more complex environmental treatments. This study provides a systematic transcriptomic characterization of the *GmHSP100* family. Future research should employ genome editing (e.g., CRISPR/Cas9), protein–protein interaction assays, and transgenic complementation to dissect the biochemical functions, regulatory networks, and agronomic contributions of these candidate genes. Such studies will clarify the molecular mechanisms by which the *GmHSP100* family enhances stress resilience and supports molecular breeding for improved abiotic stress tolerance in soybean.

## 5. Conclusions

This study systematically characterized the *GmHSP100* gene family in soybean and identified 13 family members, which were phylogenetically clustered into three distinct subfamilies: *CLPB*, *CLPC*, and *CLPD*. The expansion of the *GmHSP100* family was primarily driven by segmental duplication events, and the family exhibits high evolutionary conservation across legume species. All GmHSP100 proteins harbor the typical AAA+ ATPase core domain and are predicted to localize to the cytoplasm, chloroplasts, or mitochondria, implying that they perform compartment-specific protein quality control functions to maintain cellular homeostasis. *GmHSP100* family members exhibited pronounced tissue- and cell-type-specific expression patterns, with several members highly expressed in dividing cells of roots and nodules, indicating their potential involvement in multiple regulatory processes during plant growth and development. Furthermore, the family genes show clear time-dependent induction under heat and salt stress, with coordinated but diversified transcriptional responses. Notably, key genes, including *GmHSP101-2*, *GmCLPB4*, *GmCLPC2*, *GmCLPC3*, and *GmCLPD1*, are strongly and stably induced by both stresses, representing high-potential targets for breeding applications aimed at enhancing stress tolerance in soybean. Collectively, this study establishes a comprehensive theoretical framework for understanding the evolutionary characteristics, structural conservation, and functional divergence of the soybean *GmHSP100* family in abiotic stress adaptation. It also provides reliable candidate genes and a molecular basis for the molecular breeding of heat- and salt-tolerant soybean varieties.

## Figures and Tables

**Table 1:** Primer sequences used in this study.

Primer	Forward Primer (5′-3′)	Reverse Primer (5′-3′)	Purpose
*q-GmHSP101-1*	CCTTGAAAGGAAGAGAATGCAGC	AGTTCTTTCCGCACTTCAACA	RT-qPCR
*q-GmHSP101-2*	ACAACACTCTGTTTCGCGGT	CCACACTATAAGGCCTCCGC	
*q-GmCLPB3-1*	AGTTCACAGAGATGGCGTGG	AGCAAGCCCATTCTTCTGCT	
*q-GmCLPB3-2*	GCCAACAGAAACAACGGCAT	CGCGTTCTCGAAGACCCATT	
*q-GmCLPB3-3*	CCACGTCGTCGTTTTCTCTC	GGGAAATGGGTTTTGCGAATGA	
*q-GmCLPB3-4*	TGTGCCCAAAGAGTCCACAT	GCGGTCTAGAGGCTGGAAAA	
*q-GmCLPB4*	CAAGAGGGTGGTTGGTCAGG	GCCTTGGCAAGCTCAGTTTT	
*q-GmCLPC1*	CAGGTTTCTCAGGGCTTCGT	ATCTGGTAGCTCTTGCTCGC	
*q-GmCLPC2*	AGCCATGATTCGGCTTGACA	GGTCAGTTGACCACCCTCAG	
*q-GmCLPC3*	CAGGTTTCTCAGGGCTTCGT	TCTGGTAGCTCTTGCTTGCC	
*q-GmCLPC4*	AAAGTGCCAGAGCCAACTGT	ACAAGAGCATCATCTGTGTAATGA	
*q-GmCLPD1*	AACCGTTTCTCACACGTTCG	GAGAACTATAGGGGCGCGTG	
*q-GmCLPD2*	CGTTCCTCCGATGGGTTTCT	ACTCGCTTTGCTACCGTCAT	
*q-GmActin*	AAGCTGTTCTCTCCTTGTACGCC	GCACAGTGTGAGACACACCATCA	

**Table 2:** Basic physicochemical properties of the GmHSP100 family members.

Protein	Gene ID	Number of Amino Acid	Molecular Weight	Theoretical pI	Instability Index	Aliphatic Index	Grand Average of Hydropathicity	Predicted Location
GmCLPB3-1	*Glyma.08G242100*	974	109,756.12	6.34	40.18	90.12	−0.445	chloroplast
GmCLPB3-3	*Glyma.18G264400*	974	109,768.21	6.1	39.46	91.33	−0.428	chloroplast
GmCLPB3-2	*Glyma.13G058600*	978	110,159.38	6.26	37.38	90.24	−0.448	chloroplast
GmCLPB3-4	*Glyma.19G027900*	978	110,200.47	6.2	37.57	90.24	−0.451	chloroplast
GmCLPB4	*Glyma.04G062200*	974	109,221.71	7.01	40.44	92.61	−0.402	mitochondrion
GmHSP101-1	*Glyma.05G022200*	911	101,334.69	5.93	36.32	96.97	−0.393	cytoplasm
GmHSP101-2	*Glyma.06G202200*	753	83,392.05	6.18	34.9	97	−0.384	cytoplasm
GmCLPD1	*Glyma.04G203300*	946	103,760.96	8.81	36.68	98.55	−0.162	chloroplast
GmCLPD2	*Glyma.06G162200*	950	104,244.48	8.57	38.62	97.12	−0.173	chloroplast
GmCLPC1	*Glyma.04G200400*	922	102,553.68	6.16	42.06	94.13	−0.349	chloroplast
CmCLPC3	*Glyma.06G165200*	922	102,497.57	6.09	40.41	93.81	−0.352	chloroplast
GmCLPC2	*Glyma.05G201100*	919	102,367.45	6.35	42.82	91.78	−0.39	chloroplast
CmCLPC4	*Glyma.08G008400*	919	102,169.2	6.37	44.29	92.63	−0.378	chloroplast

## Data Availability

The original contributions presented in the study are included in the article/Supplementary Materials. Further inquiries can be directed to the corresponding authors.

## Supplementary Materials

The following supporting information can be downloaded at: https://www.mdpi.com/article/10.3390/genes17060608/s1, Table S1: The amino acid sequences HSP100s in Arabidopsis, rice, and soybean; Table S2: Gene ID of GmHSP100s in the cultivar of Zhonghuang 13 and W82.
